# Supplementary material for: Pyruvate Dehydrogenase Kinase 1 inhibition mediated oxidative phosphorylation enhancement in cartilage promotes osteoarthritis progression
Source: BMC Musculoskelet Disord. 2023 Jul 20;24:597. doi: 10.1186/s12891-023-06585-6 (PMC10357736; doi:10.1186/s12891-023-06585-6)
Supplement: Supplementary file 1 — Additional file 1. [file 12891_2023_6585_MOESM1_ESM.zip › BP.Gsea.1653623667859/GOBP_RIBOSOMAL_SMALL_SUBUNIT_BIOGENESIS.html]

Details for gene set GOBP\_RIBOSOMAL\_SMALL\_SUBUNIT\_BIOGENESIS[GSEA]

|  || Dataset | 55457\_collapsed\_to\_symbols.cls.cls #OA\_versus\_normal.cls.cls #OA\_versus\_normal\_repos |
| Phenotype | cls.cls#OA\_versus\_normal\_repos |
| Upregulated in class | normal |
| GeneSet | GOBP\_RIBOSOMAL\_SMALL\_SUBUNIT\_BIOGENESIS |
| Enrichment Score (ES) | -0.47448802 |
| Normalized Enrichment Score (NES) | -1.864477 |
| Nominal p-value | 0.0 |
| FDR q-value | 0.06429095 |
| FWER p-Value | 0.847 |
Table: GSEA Results Summary

  

Fig 1: Enrichment plot: GOBP\_RIBOSOMAL\_SMALL\_SUBUNIT\_BIOGENESIS      
 Profile of the Running ES Score & Positions of GeneSet Members on the Rank Ordered List

  

| SYMBOL | TITLE | RANK IN GENE LIST | RANK METRIC SCORE | RUNNING ES | CORE ENRICHMENT || 1 | XRCC5 | X-ray repair cross complementing 5 [Source:HGNC Symbol;Acc:HGNC:12833] | 1018 | 0.513 | -0.0542 | No |
| 2 | GTF2H5 | general transcription factor IIH subunit 5 [Source:HGNC Symbol;Acc:HGNC:21157] | 1032 | 0.511 | -0.0302 | No |
| 3 | MRPS11 | mitochondrial ribosomal protein S11 [Source:HGNC Symbol;Acc:HGNC:14050] | 1305 | 0.457 | -0.0290 | No |
| 4 | MRPS7 | mitochondrial ribosomal protein S7 [Source:HGNC Symbol;Acc:HGNC:14499] | 1650 | 0.406 | -0.0359 | No |
| 5 | EMG1 | EMG1 N1-specific pseudouridine methyltransferase [Source:HGNC Symbol;Acc:HGNC:16912] | 1688 | 0.401 | -0.0192 | No |
| 6 | TBL3 | transducin beta like 3 [Source:HGNC Symbol;Acc:HGNC:11587] | 2154 | 0.344 | -0.0386 | No |
| 7 | TSR2 | TSR2 ribosome maturation factor [Source:HGNC Symbol;Acc:HGNC:25455] | 2924 | 0.258 | -0.0859 | No |
| 8 | ERCC2 | "ERCC excision repair 2, TFIIH core complex helicase subunit [Source:HGNC Symbol;Acc:HGNC:3434]" | 3544 | 0.198 | -0.1244 | No |
| 9 | RIOK2 | RIO kinase 2 [Source:HGNC Symbol;Acc:HGNC:18999] | 3615 | 0.192 | -0.1204 | No |
| 10 | SNU13 | small nuclear ribonucleoprotein 13 [Source:HGNC Symbol;Acc:HGNC:7819] | 3795 | 0.178 | -0.1257 | No |
| 11 | RPS27L | ribosomal protein S27 like [Source:HGNC Symbol;Acc:HGNC:18476] | 3882 | 0.166 | -0.1242 | No |
| 12 | LSM6 | "LSM6 homolog, U6 small nuclear RNA and mRNA degradation associated [Source:HGNC Symbol;Acc:HGNC:17017]" | 4019 | 0.154 | -0.1273 | No |
| 13 | RIOK3 | RIO kinase 3 [Source:HGNC Symbol;Acc:HGNC:11451] | 4206 | 0.137 | -0.1351 | No |
| 14 | ABT1 | activator of basal transcription 1 [Source:HGNC Symbol;Acc:HGNC:17369] | 4783 | 0.088 | -0.1756 | No |
| 15 | UTP3 | UTP3 small subunit processome component [Source:HGNC Symbol;Acc:HGNC:24477] | 4934 | 0.077 | -0.1835 | No |
| 16 | METTL17 | methyltransferase like 17 [Source:HGNC Symbol;Acc:HGNC:19280] | 5673 | 0.018 | -0.2401 | No |
| 17 | RPS19 | ribosomal protein S19 [Source:HGNC Symbol;Acc:HGNC:10402] | 5952 | -0.002 | -0.2617 | No |
| 18 | NOL11 | nucleolar protein 11 [Source:HGNC Symbol;Acc:HGNC:24557] | 5984 | -0.003 | -0.2639 | No |
| 19 | ERAL1 | Era like 12S mitochondrial rRNA chaperone 1 [Source:HGNC Symbol;Acc:HGNC:3424] | 6158 | -0.017 | -0.2766 | No |
| 20 | NGDN | neuroguidin [Source:HGNC Symbol;Acc:HGNC:20271] | 6370 | -0.033 | -0.2914 | No |
| 21 | DCAF13 | DDB1 and CUL4 associated factor 13 [Source:HGNC Symbol;Acc:HGNC:24535] | 7459 | -0.115 | -0.3705 | No |
| 22 | TSR3 | TSR3 ribosome maturation factor [Source:HGNC Symbol;Acc:HGNC:14175] | 7676 | -0.133 | -0.3808 | No |
| 23 | NOL10 | nucleolar protein 10 [Source:HGNC Symbol;Acc:HGNC:25862] | 7880 | -0.146 | -0.3894 | No |
| 24 | RPP40 | ribonuclease P/MRP subunit p40 [Source:HGNC Symbol;Acc:HGNC:20992] | 8318 | -0.179 | -0.4147 | No |
| 25 | RPS21 | ribosomal protein S21 [Source:HGNC Symbol;Acc:HGNC:10409] | 8394 | -0.185 | -0.4115 | No |
| 26 | UTP6 | UTP6 small subunit processome component [Source:HGNC Symbol;Acc:HGNC:18279] | 8638 | -0.203 | -0.4205 | No |
| 27 | UTP25 | UTP25 small subunit processor component [Source:HGNC Symbol;Acc:HGNC:28440] | 8699 | -0.209 | -0.4149 | No |
| 28 | RPSA | ribosomal protein SA [Source:HGNC Symbol;Acc:HGNC:6502] | 8866 | -0.222 | -0.4170 | No |
| 29 | BYSL | bystin like [Source:HGNC Symbol;Acc:HGNC:1157] | 9277 | -0.254 | -0.4365 | No |
| 30 | RPS25 | ribosomal protein S25 [Source:HGNC Symbol;Acc:HGNC:10413] | 9282 | -0.254 | -0.4244 | No |
| 31 | HEATR1 | HEAT repeat containing 1 [Source:HGNC Symbol;Acc:HGNC:25517] | 9339 | -0.259 | -0.4161 | No |
| 32 | RCL1 | RNA terminal phosphate cyclase like 1 [Source:HGNC Symbol;Acc:HGNC:17687] | 9586 | -0.282 | -0.4214 | No |
| 33 | PRKDC | "protein kinase, DNA-activated, catalytic subunit [Source:HGNC Symbol;Acc:HGNC:9413]" | 9632 | -0.286 | -0.4110 | No |
| 34 | RRP7A | ribosomal RNA processing 7 homolog A [Source:HGNC Symbol;Acc:HGNC:24286] | 10449 | -0.367 | -0.4565 | Yes |
| 35 | RPS8 | ribosomal protein S8 [Source:HGNC Symbol;Acc:HGNC:10441] | 10525 | -0.376 | -0.4440 | Yes |
| 36 | RPS14 | ribosomal protein S14 [Source:HGNC Symbol;Acc:HGNC:10387] | 10558 | -0.379 | -0.4279 | Yes |
| 37 | RPS7 | ribosomal protein S7 [Source:HGNC Symbol;Acc:HGNC:10440] | 10608 | -0.384 | -0.4130 | Yes |
| 38 | NOP14 | NOP14 nucleolar protein [Source:HGNC Symbol;Acc:HGNC:16821] | 10747 | -0.398 | -0.4042 | Yes |
| 39 | NAT10 | N-acetyltransferase 10 [Source:HGNC Symbol;Acc:HGNC:29830] | 10998 | -0.429 | -0.4027 | Yes |
| 40 | RRS1 | ribosome biogenesis regulator 1 homolog [Source:HGNC Symbol;Acc:HGNC:17083] | 11045 | -0.437 | -0.3849 | Yes |
| 41 | RPL38 | ribosomal protein L38 [Source:HGNC Symbol;Acc:HGNC:10349] | 11122 | -0.446 | -0.3690 | Yes |
| 42 | RPS5 | ribosomal protein S5 [Source:HGNC Symbol;Acc:HGNC:10426] | 11206 | -0.458 | -0.3531 | Yes |
| 43 | WDR3 | WD repeat domain 3 [Source:HGNC Symbol;Acc:HGNC:12755] | 11429 | -0.492 | -0.3463 | Yes |
| 44 | DDX52 | DExD-box helicase 52 [Source:HGNC Symbol;Acc:HGNC:20038] | 11484 | -0.499 | -0.3261 | Yes |
| 45 | PWP2 | PWP2 small subunit processome component [Source:HGNC Symbol;Acc:HGNC:9711] | 11604 | -0.516 | -0.3101 | Yes |
| 46 | UTP20 | UTP20 small subunit processome component [Source:HGNC Symbol;Acc:HGNC:17897] | 11656 | -0.526 | -0.2883 | Yes |
| 47 | RPS6 | ribosomal protein S6 [Source:HGNC Symbol;Acc:HGNC:10429] | 11662 | -0.526 | -0.2630 | Yes |
| 48 | RPS15 | ribosomal protein S15 [Source:HGNC Symbol;Acc:HGNC:10388] | 11671 | -0.527 | -0.2378 | Yes |
| 49 | WDR46 | WD repeat domain 46 [Source:HGNC Symbol;Acc:HGNC:13923] | 11683 | -0.529 | -0.2128 | Yes |
| 50 | RPS16 | ribosomal protein S16 [Source:HGNC Symbol;Acc:HGNC:10396] | 11755 | -0.541 | -0.1918 | Yes |
| 51 | RPS27 | ribosomal protein S27 [Source:HGNC Symbol;Acc:HGNC:10416] | 12071 | -0.612 | -0.1864 | Yes |
| 52 | KRI1 | KRI1 homolog [Source:HGNC Symbol;Acc:HGNC:25769] | 12074 | -0.613 | -0.1566 | Yes |
| 53 | RPS17 | ribosomal protein S17 [Source:HGNC Symbol;Acc:HGNC:10397] | 12130 | -0.626 | -0.1302 | Yes |
| 54 | RPS24 | ribosomal protein S24 [Source:HGNC Symbol;Acc:HGNC:10411] | 12223 | -0.652 | -0.1055 | Yes |
| 55 | BMS1 | BMS1 ribosome biogenesis factor [Source:HGNC Symbol;Acc:HGNC:23505] | 12403 | -0.707 | -0.0849 | Yes |
| 56 | RPS28 | ribosomal protein S28 [Source:HGNC Symbol;Acc:HGNC:10418] | 12526 | -0.761 | -0.0572 | Yes |
| 57 | NPM1 | nucleophosmin 1 [Source:HGNC Symbol;Acc:HGNC:7910] | 12530 | -0.762 | -0.0201 | Yes |
| 58 | TSR1 | TSR1 ribosome maturation factor [Source:HGNC Symbol;Acc:HGNC:25542] | 12814 | -0.999 | 0.0067 | Yes |
Table: GSEA details [plain text format]

  

Fig 2: GOBP\_RIBOSOMAL\_SMALL\_SUBUNIT\_BIOGENESIS      
 Blue-Pink O' Gram in the Space of the Analyzed GeneSet

  

Fig 3: GOBP\_RIBOSOMAL\_SMALL\_SUBUNIT\_BIOGENESIS: Random ES distribution      
 Gene set null distribution of ES for **GOBP\_RIBOSOMAL\_SMALL\_SUBUNIT\_BIOGENESIS**
